# Systemic Inflammatory Response and Elevated Tumour Markers Predict Worse Survival in Resectable Pancreatic Ductal Adenocarcinoma

**DOI:** 10.1371/journal.pone.0163064

**Published:** 2016-09-15

**Authors:** Aino Salmiheimo, Harri Mustonen, Ulf-Håkan Stenman, Pauli Puolakkainen, Esko Kemppainen, Hanna Seppänen, Caj Haglund

**Affiliations:** 1 Department of Surgery, University of Helsinki and Helsinki University Hospital, Helsinki, Finland; 2 Department of Clinical Chemistry, University of Helsinki and Helsinki University Hospital, Helsinki, Finland; 3 Research Programs Unit, Translational Cancer Biology Program, University of Helsinki, Helsinki, Finland; University of North Carolina at Chapel Hill School of Medicine, UNITED STATES

## Abstract

**Background:**

Estimation of the prognosis of resectable pancreatic ductal adenocarcinoma (PDAC) currently relies on tumour-related factors such as resection margins and on lymph-node ratio (LNR) both inconveniently available only postoperatively. Our aim was to assess the accuracy of preoperative laboratory data in predicting PDAC prognosis.

**Methods:**

Collection of laboratory and clinical data was retrospective from 265 consecutive patients undergoing surgery for PDAC at Helsinki University Hospital. Cancer-specific survival assessment utilized Kaplan-Meier analysis, and independent associations between factors were by the Cox regression model.

**Results:**

During follow-up, 76% of the patients died of PDAC, with a median survival time of 19.6 months. In univariate analysis, CRP, albumin, CEA, and CA19-9 were significantly associated with postoperative cancer-specific survival. In multivariate analysis, taking into account age, gender, LNR, resection margins, tumour status, and adjuvant chemotherapy, the preoperative biomarkers independently associated with adverse prognosis were hypoalbuminemia (< 36 g/L, hazard ratio (HR) 1.56, 95% confidence interval (CI) 1.10–2.19, *p* = 0.011), elevated CRP (> 5 mg/L, HR 1.44, 95% CI 1.03–2.02, *p* = 0.036), CEA (> 5 μg/L, HR 1.60, 95% CI 1.07–2.53, *p* = 0.047), and CA19-9 (≥555 kU/L, HR 1.91, 95% CI 1.18–3.08, *p* = 0.008).

**Conclusion:**

For patients with resectable PDAC, preoperative CRP, along with albumin and tumour markers, is useful for predicting prognosis.

## Introduction

Pancreatic ductal adenocarcinoma (PDAC) is worldwide the fourth most common cause of cancer death. It has an appalling 5-year overall survival rate of < 8% [1,2], and the only possibility of cure is early radical surgery. Unfortunately, less than 10% of the patients are diagnosed at a localized stage due to this cancer’s tendency to metastasize aggressively; even at its localized stage, the 5-year survival rate is only 10–27% [1–3]. Moreover, pancreatic surgery itself is associated with rather high morbidity and mortality. Factors predicting the survival of pancreatic cancer patients include TNM stage, resection margin, lymph-node ratio (LNR, metastatic lymph nodes divided by number of lymph nodes analysed), vascular invasion, and differentiation grade, each of these, however, typically revealed only during or after surgery [4–6].

Nowadays, evidence is increasing as to an association between cancer progression and inflammation [7–9]. A cancer-related systemic inflammatory response (SIR), indicated by elevated concentrations of circulating acute phase proteins such as C-reactive protein (CRP), is in various cancer forms associated with worse prognosis [10–12]. In pancreatic cancer, the association between CRP and prognosis has been under study mostly in patients receiving palliative chemotherapy or in small patient groups [13,14]. Albumin is the most abundant protein in human serum. Low concentrations of serum albumin (hypoalbuminemia) indicates poor nutritional status and low performance status but albumin may also decrease due to many other conditions such as systemic inflammation [15].

The Glasgow prognostic score (GPS), originally developed in a cohort of patients with non-small cell lung cancer, combines elevated CRP and low albumin values to estimate prognosis [16]. Later, because some studies failed to show hypoalbuminemia to be an independent predictor of survival, the GPS was modified by emphasizing elevated CRP [17]. This modified Glasgow prognostic score (mGPS) predicts cancer survival independently of tumour site [18].

The biomarker most commonly serving for diagnosis, follow-up, and prognostic evaluation of pancreatic cancer is the serum tumour marker CA19-9 [19–22]. Carcinoembryonic antigen (CEA) at diagnosis has a lower sensitivity and specificity for PDAC than does CA19-9 [22].

The aim of this study was to evaluate preoperative CRP and albumin in the estimation of postoperative survival of patients with resectable PDAC. We compared these markers with prognostic clinico-pathological characteristics and tumour markers. Improving the accuracy of preoperative estimation of prognosis could aid in the selection of patients for surgery, especially in borderline cases.

## Patients and Methods

We collected data from all 292 patients undergoing surgery for histologically verified pancreatic ductal adenocarcinoma (PDAC) at Helsinki University Hospital (HUH) between 1 January, 2000 and 31 March, 2013. A pathologist specialized in pancreatology has retrospectively re-examined the slides with tumour specimens to verify the diagnosis, and to determine the resection margin, when possible. Patients undergoing emergency surgery, those who died of surgery-related complications, and those with ongoing infection, auto-immune disease, or immunosuppressive medication at the time of surgery, totalling 7, were excluded. We included only patients undergoing surgery with curative intent and excluded the 20 patients whose surgery revealed metastatic spread or otherwise unresectable disease. The operations included distal pancreatic resection, Whipple-Kausch pancreaticoduodenectomy, pylorus-preserving pancreaticoduodenectomy, and total pancreatectomy. Some patients received pre- and postoperative oncological treatment: preoperatively either gemcitabine with or without radiotherapy, postoperatively gemcitabine, capecitabine, or a combination of gemcitabine and cisplatin or capecitabine either as adjuvant therapy or later as palliative treatment for those patients who had no adjuvant therapy but received palliative chemotherapy only after disease progression was found postoperatively (Table 1). We collected data on case-report forms linked to an Access^®^ database and converted it for analysis with the IBM Statistical Package for Social Sciences (SPSS) Statistics 22. This study applied the Reporting Recommendations for Tumour Marker Prognostic Studies (REMARK) criteria [23]. The end of follow-up was 31 March 2015, with a minimum follow-up of 2 years.

**Table 1:** Patient characteristics and survival time.

Total n = 265	n (%)	Median survivalmonths (95% CI)	*p*
**Sex**			
Male	150 (56.6)	25.4 (18.9–31.9)	0.453
Female	115 (43.4)	26.9 (23.8–30.0)	
**Age**			
< 60 years	70 (26.4)	27.4 (19.8–35.0)	0.177
60–64 years	57 (21.5)	25.7 (17.4–34.1)	
65–70 years	59 (22.3)	26.3 (16.8–35.9)	
> 70 years	79 (29.8)	26.1 (19.2–32.9)	
**Margin involvement**			
R0	207 (78.1)	30.0 (25.1–35.0)	**< 0.001**
R1	42 (15.8)	18.3 (7.6–28.9)	
Data missing	16 (6.0)		
**T status**			
T1	21 (7.9)	27.2 (15.9–38.5)	**0.003**
T2	64 (24.2)	36.0 (22.3–49.7)	
T3	166 (62.6)	22.0 (17.7–26.3)	
T4	8 (3.0)	14.7 (7.4–22.0)	
Data missing	6 (2.3)		
**Nodal metastases**			
N0	94 (35.5)	33.6 (30.4–36.8)	**0.001**
N1	169 (63.8)	21.5 (17.3–25.7)	
Data missing	2 (0.8)		
**LNR**			
N0	94 (35.5)	33.6 (30.4–36.8)	**< 0.001**
N1, ≤ 0.2	123 (46.4)	25.7 (17.9–33.6)	
N1, > 0.2	42 (15.8)	13.6 (7.7–19.4)	
Data missing	6 (2.3)		
**Tumour location**			
Head	230 (86.8)	26.0 (21.8–30.1)	0.734
Tail	13 (4.9)	34.1 (9.4–58.8)	
Body	19 (7.2)	36.0 (10.3–61.7)	
Data missing	3 (1.1)		
**Tumour size**			
≤ 30 mm	135 (51.0)	30.1 (24.8–35.4)	**0.007**
> 30 mm	120 (45.0)	20.5 (15.6–25.4)	
Data missing	10 (4.0)		
**ERCP and stenting**			
No ERCP	89 (33.6)	27.1 (16.6–37.6)	0.640
ERCP + stent	169 (63.8)	26.4 (22.2–30.7)	
Data missing	7 (2.6)		
**Preoperative chemo-/radiotherapy**			
None	218 (82.3)	25.7 (20.8–30.6)	0.573
Yes	46 (17.4)	30.0 (23.7–36.4)	
Data missing	1 (0.4)		
**Postoperative chemo-/radiotherapy**			
None	86 (32.5)	21.9 (14.5–29.3)	0.058
Adjuvant	139 (52.5)	30.1 (24.1–36.0)	
Palliative	34 (12.8)	23.9 (15.4–32.5)	
Data missing	6 (2.3)		
**Cause of death **			
Pancreatic cancer	202 (76.2)	–	
Other	10 (3.8)	–	
Alive	53 (20.0)	–	

Median survival time was assessed by Kaplan-Meier analysis and significance by log-rank test.

### Patient characteristics

Patients totalled 265 (characteristics are summarized in Table 1). Mean age for the operation was 65 years (standard deviation (SD) ± 8.6), ranging from 39 to 86 years. Median overall follow-up was 25.2 months. At the end of follow-up, 202 (76%) study patients had died of PDAC, with a median survival of 19.6 months (range: 1.1 month to 9.3 years), 53 (20%) patients were still alive (follow-up range: 2.0 to 14.1 years), and 10 (4%) patients had died of other causes.

### High-sensitivity CRP

CRP levels were determined from 230 plasma samples prospectively collected with informed consent for research purposes before surgery and stored frozen at -80°C until assayed [24]. Plasma CRP was determined by a time-resolved immunofluorometric assay performed in microtitration plates using a monoclonal CRP antibody (anti-hCRP, code 6405, Medix Biochemica, Espoo, Finland). Because CRP is a pentamer, the same antibody served to capture CRP and served also as a tracer. The antibody was diluted to a concentration of 5 μg/ml in phosphate-buffered saline (PBS) and coated onto the solid phase by incubation of 200 μl in each well overnight. The same antibody, labelled with europium chelate, served as a tracer. Calibrators (Labquality, Helsinki, Finland) covered the concentration range 3 μg/L–300 μg/L. The samples were diluted 100-fold before the assay; thus the measuring range was 0.3 mg/L–30 mg/L. Imprecision was < 10% over the whole assay range.

### Other laboratory analyses

All laboratory methods, except for the CRP assay, were standard methods of the clinical laboratory of HUH. In the statistical analyses we used predominantly standard cut-off values recommended by the manufacturer and used in clinical practice in our hospital. Hypoalbuminemia, elevated CRP, and CA19-9 we further divided, in order to explore whether survival worsens with greater divergence from the normal values. Laboratory data collected from patient records included leukocytes (n = 265), albumin (259), platelets (262), and bilirubin (262), available up to 5 days preoperatively, as well as CA19-9 (255) and CEA (252), which were determined up to 2 months preoperatively, always using the latest value available before the day of surgery. The vast majority of laboratory measurements were determined the day before surgery. According to Finnish guidelines, any albumin level < 36 g/L was considered hypoalbuminemic, except when calculating the GPS.

### The Glasgow prognostic score

When calculating the GPS, either an elevated CRP (> 10 mg/L) or a low albumin level (< 35 g/L) received a score of 1. Patients having both elevated CRP and hypoalbuminemia scored GPS 2, and those with normal CRP < 10 mg/L and albumin > 35 g/L scored GPS 0 [16]. We also calculated the modified Glasgow prognostic score (mGPS), according to which, mGPS scored 0 if CRP was normal (< 10 mg/L), irrespective of albumin level; mGPS score 1 represented elevated CRP (> 10 mg/L), and score 2 represented elevated CRP combined with low albumin [18]. If either of these values was missing, we excluded that patient from GPS- and mGPS analysis.

### Statistics

We conducted statistical analyses with IBM SPSS 22, and calculated associations between continuous variables by two-tailed Spearman’s correlations, normality by the Kolmogorov-Smirnov test, and differences in distributions by the Mann-Whitney U- or the Kruskall-Wallis test. The Jonckheere-Terpstra test allowed calculation of the significance of differences in medians of laboratory tests by survival time (ordinal scale). Survival time was defined from pancreatic surgery to disease-specific death; if no events had occurred, each patient was censored at the last follow-up date. Kaplan-Meier analysis and the log-rank test served for survival estimation, and the Cox proportional hazards model for the multivariate analysis. We included in the multivariate analysis variables with a *p* value < 0.1 in univariate analysis, and also the clinically significant variables age and sex. The multivariate analyses included only those 189 patients with no missing values. Interactions were considered, but no significant interactions emerged. A *p* value < 0.05 indicated the limit for statistical significance.

### Ethics

This study followed the ethical, medical, and legal guidelines of Finland and the Declaration of Helsinki. Approval came from the Helsinki University Hospital Ethics Committee and the National Supervisory Authority for Welfare and Health (Valvira). Blood samples for research purposes were taken only after written informed consent from the patients. The guidelines of HUH and National Data Protection ensured the confidentiality of patient information.

## Results

### Laboratory results

A summary of preoperative laboratory results appears in Table 2 and their associations with clinico-pathological features in Table 3. Median preoperative CRP was 3.7 mg/L (inter quartile range (IQR) 1.7–10.1 mg/L). Patients who died of PDAC had a higher median CRP 4.9 mg/L (IQR 1.8–13.1 mg/L) than the median CRP of 1.9 mg/L (IQR 1.2–3.6 mg/L) (*p* < 0.001) of patients alive throughout follow-up. Furthermore, the higher the CRP, the shorter the survival time (Table 3). CRP showed no association with T status, LNR, nor tumour size (Table 3). Administration of neoadjuvant therapy had no significant association with CRP (*p* = 0.538).

**Table 2:** Preoperative biomarkers' association with median survival time and the univariate analysis.

Factors	n	Median survival months (95% CI)	*p*(survival time)	HR (95% CI)	*p*(HR)	Median (IQR)
**CRP**						3.7 (8.4)
≤ 5.0 mg/L	130	31.8 (25.7–37.9)	**< 0.001**	1	**< 0.001**	
5.1–15.0 mg/L	58	26.3 (21.5–31.2)		1.4 (1.02–2.04)	**0.040**	
> 15.0 mg/L	42	14.4 (4.4–24.4)		2.3 (1.6–3.3)	**< 0.001**	
Missing	35					
**Albumin**						37.0 (4.3)*
≤ 30.0 g/L	16	10.0 (2.9–17.0)	**< 0.001**	3.0 (1.8–5.0)	**< 0.001**	
30.1–35.9 g/L	83	18.1 (13.1–23.0)		1.6 (1.2–2.2)	**0.001**	
≥ 36.0 g/L	160	31.7 (26.6–36.8)		1	**< 0.001**	
Missing	6					
**CA19-9**						133.5 (501.0)
≤ 37 kU/L	81	33.5 (25.9–41.1)	**< 0.001**	1	**< 0.001**	
38–554 kU/L	114	26.0 (20.0–32.0)		1.5 (1.01–2.0)	**0.022**	
≥ 555 kU/L	60	16.1 (7.4–24.7)		2.1 (1.5–3.1)	**< 0.001**	
Missing	10					
**CEA**						2.7 (2.7)
≤ 5.0 μg/L	205	27.4 (23.7–31.1)	**0.016**	1		
> 5.0 μg/L	47	14.4 (22.7–30.0)		1.5 (1.1–2.2)	**0.017**	
Missing	13					
**Bilirubin**						16.0 (25.0)
≤ 20 g/L	154	26.4 (22.7–30.1)	0.153	1		
> 20 g/L	108	23.0 (14.6–31.5)		1.2 (0.9–1.6)	0.154	
Missing	3					
**Platelets**						235.5 (101.0)
150–360 E9/L	211	26.9 (23.5–30.3)	0.642	1	0.644	
<150 E9/L	21	23.9 (0.0–54.5)		0.8 (0.5–1.4)	0.378	
>360 E9/L	30	20.4 (5.1–35.6)		1.1 (0.7–1.6)	0.817	
Missing	3					
**Leukocytes**						6.1 (2.7)
3.4–8.2 E9/L	204	26.3 (22.2–30.5)	0.685	1	0.686	
< 3.4 E9/L	8	23.9 (0.0–50.9)		0.8 (0.4–1.9)	0.669	
> 8.2 E9/L	53	25.5 (16.5–34.4)		1.1 (0.8–1.6)	0.476	
Missing	0					
**GPS**						–
0	136	31.7 (25.9–37.5)	**< 0.001**	1	**< 0.001**	
1	59	21.5 (13.6–29.4)		1.7 (1.2–2.4)	**0.004**	
2	30	14.4 (2.8–26.0)		2.4 (1.6–3.6)	**< 0.001**	
Missing	40					
**mGPS**						–
0	172	29.6 (25.1–34.0)	**< 0.001**	1	**< 0.001**	
1	23	24.9 (17.0–32.8)		1.6 (0.99–2.6)	0.053	
2	30	14.4 (2.8–26.0)		2.2 (1.4–3.2)	**< 0.001**	
Missing	40					

Abbreviations: HR, hazard ratio; CI, confidence interval; GPS, Glasgow prognostic score; mGPS, modified Glasgow prognostic score; IQR, inter quartile range

* Mean albumin (standard deviation)

Preoperative biomarkers' association with median survival time (months) was assessed with Kaplan- Meier analysis. Univariate analysis of the hazard ratios (HR) was by Cox regression model.

**Table 3:** Associations of preoperative biomarkers with patient- and tumour-related factors.

	CRP (mg/L)		Albumin (g/L)		Ca19-9 (kU/L)		CEA (μg/L)	
	Median (IQR)	*p*	Mean (SD)	*p*	Median (IQR)	*p*	Median (IQR)	*p*
**Sex**								
Male	3.5 (8.2)	0.853	37.1 (4.5)	0.421	133.0 (511)	0.727	2.7 (3.1)	0.724
Female	4.4 (8.2)		36.7 (4.0)		129.5 (488)		2.7 (2.4)	
**Age**								
< 65	3.6 (7.2)	0.220	37.5 (4.4)	**0.033**	102.0 (452)	0.095	2.6 (3.2)	0.655
≥ 65	3.9 (9.3)		36.4 (4.2)		140.0 (561)		2.7 (2.5)	
**T status**								
T1–T2	3.2 (6.5)	0.073	37.8 (4.1)	**0.026**	63.0 (222)	**0.007**	2.5 (2.3)	**0.023**
T3–T4	4.5 (9.5)		36.5 (4.4)		185.5 (558)		2.8 (2.9)	
**LNR**								
N0	3.1 (7.4)	0.652	37.0 (4.1)	0.499	79.0 (414)	**0.018**	2.7 (2.6)	0.109
N1, ≤ 0.2	3.6 (7.8)		36.9 (4.6)		129.0 (428)		2.7 (2.7)	
N1, > 0.2	4.8 (12.2)		36.7 (4.1)		274.5 (1612)		3.2 (2.9)	
**Tumour size**								
≤ 30 mm	3.23 (7.3)	0.072	37.4 (4.2)	**0.018**	65.5 (333)	**< 0.001**	2.6 (2.3)	**0.033**
>30 mm	4.5 (11.7)		36.2 (4.4)		223 (839)		3.1 (3.4)	
**Cause of death**								
PDAC	4.9 (11.3)	**< 0.001**	36.4 (4.4)	**0.001**	147.0 (570)	**0.038**	2.8 (2.6)	0.208
Other or alive	2.8 (3.5)		38.5 (3.5)		56.5 (301)		2.4 (2.5)	
**Survival time**								
< 3 months	19.5 (83.3)	**0.001**	30.3 (4.0)	**< 0.001**	780 (3283)	**< 0.001**	3.9 (3.1)	**0.010**
3–12 months	6.2 (14.8)		35.9 (4.4)		336 (972)		3.4 (4.5)	
12–24 months	4.4 (7.8)		36.5 (3.9)		146 (606)		2.7 (2.0)	
> 24 months	2.8 (6.6)		37.8 (4.1)		69 (362)		2.5 (2.3)	

PDAC, pancreatic ductal adenocarcinoma; LNR, lymph-node ratio; IQR, inter quartile range; SD, standard deviation

Significances were determined with the Mann-Whitney U or the Jonckheere-Terpstra test (abnormal distribution) or with the T-test and One-Way ANOVA for linear contrasts (normal distribution).

Mean preoperative albumin was 37.0 g/L (SD 4.3 g/L). The majority of patients had normal albumin levels, whereas 38% had hypoalbuminemia (< 36.0 g/L). Albumin was associated with patient age, T status, and tumour size (Table 3). Patients who died of PDAC had lower mean albumin than did patients dying of other causes or alive at the end of follow-up; the shorter the survival, the lower the mean albumin (*p* = 0.001) (Table 3).

CA19-9 (median 133 kU/L, IQR 21–526 kU/L) and CEA (median 2.7 μg/L, IQR 1.8–4.4 μg/L) were associated with T status and tumour size (Table 3). Both tended to increase with shorter survival time (*p* = 0.001). CA19-9 increased also with increasing LNR status (*p* = 0.018) (Table 3). Additionally, bilirubin level and leukocyte and platelet counts were analysed but showed no association with disease-specific survival in univariate analysis (Table 3).

### Tumour-related factors

Median tumour size was 30 mm, ranging from 5 mm to 90 mm (255 tumours). Resection with radical > 1 mm margins (R0) was successful in 207 (78%) cases (data on resection margins were missing from 16 pathology reports). Lymph node metastases occurred in 169 (64%) patients, and 94 (36%) were lymph-node negative (2 patients had no lymph-node examination). Median LNR was 0.06 (IQR 0.00–0.16).

### Univariate analyses

**Fig 1:**
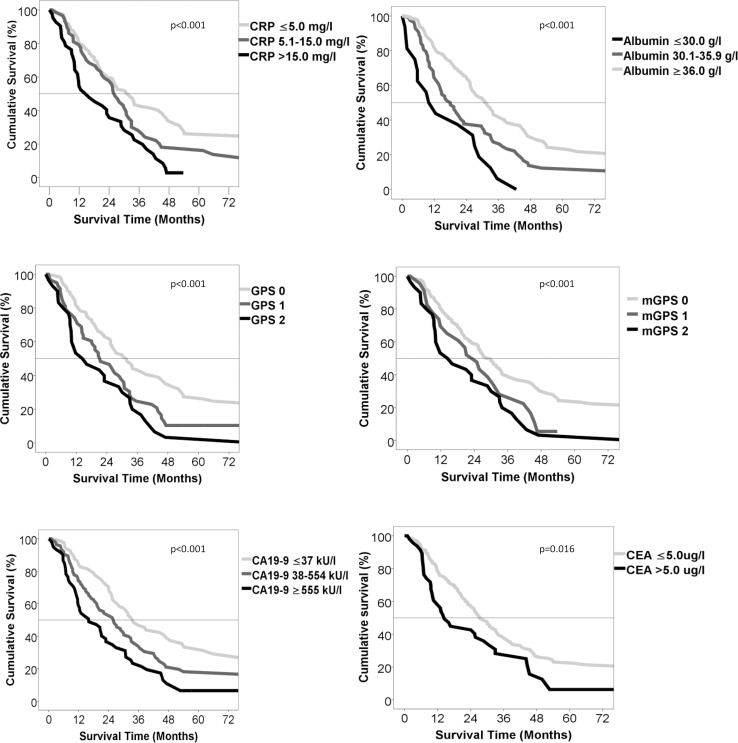
Survival curves. Effects of preoperative C-reactive protein (CRP), albumin, Glasgow prognostic score (GPS), modified GPS (mGPS), CA19-9, and CEA on disease-specific survival in patients with surgically treated pancreatic cancer. Survival curves (survival time in months after surgery) by Kaplan-Meier analysis and significance by log-rank test.

Univariate associations of clinico-pathological features and laboratory results with survival are presented in Tables 1 and 2, and survival curves in Fig 1. In Kaplan-Meier analysis, median survival time for patients with CRP 5.0 mg/L or lower was 31.8 months (95% CI 25.7–37.9) compared to 23.9 months (95% CI 19.5–28.4, *p* < 0.001) for those at over 5.0 mg/L (HR 1.7, 95% CI 1.3–2.3, *p* < 0.001). Patients suffering from hypoalbuminemia (< 36 g/L) had a significantly shorter survival time of 18.0 months (95% CI 12.7–23.2) compared to 31.7 months (95% CI 26.6–36.8) (*p* < 0.001) for those with normal albumin level (HR 1.8, 95% CI 1.3–2.4, *p* < 0.001).

We assessed the impact of both the GPS and the mGPS (emphasizing elevated CRP) in prognosis evaluation for resectable PDAC. Median survival time decreased significantly with increasing scores for both GPS and mGPS, although the hazard ratio (HR) 1.6 for mGPS 1 was not statistically significant (*p* = 0.053). The HR for GPS 1 was 1.7 (*p* = 0.004) and for GPS 2 was 2.4 (*p* < 0.001) (Table 2, Fig 1).

Elevated concentrations of CEA (> 5 μg/L), and CA19-9 (> 38 kU/L) were significantly associated in univariate analyses with worse survival (Table 2, Fig 1). Of the clinico-pathological factors, tumour size, radical resection margins, T status, nodal metastases, and LNR associated significantly with worse survival (Table 1).

### Multivariate analyses

Regarding gender, age, T status, LNR, adjuvant therapy, tumour size, and resection radicality, the biomarkers that were independently associated with adverse prognosis in multivariate analysis were low albumin (< 36 g/L, *p* = 0.011), elevated CRP (> 5 mg/L, *p* = 0.036), CEA (> 5 μg/L, *p* = 0.047), and CA19-9, but the latter only when elevated 15-fold above its reference limit (≥ 555 kU/L, *p* = 0.008) (Table 4). Administration of adjuvant therapy, R0 resection margins, and low LNR (N0 and LNR ≤ 0.2) were independently associated with more favourable prognosis. In a multivariate model including otherwise the same variables as in Table 4 but using continuous values for laboratory results, CRP (lg10) (HR 1.53, CI 95% 1.07–2.17, *p* = 0.018), CA19-9 (lg10) (HR 1.28, CI 95% 1.05–1.56, *p* = 0.015), and albumin (HR 0.95, CI 95% 0.91–0.99, *p* = 0.020) each showed a linear association with survival.

**Table 4:** Multivariate model considering clinically and statistically important patient characteristics and preoperative biomarkers (n = 189).

Factor	Description	HR (95% CI)	*p* value
Age (years)	<65/≥65	1.11 (0.78–1.58)	0.554
Sex	Male/female	1.36 (0.96–1.95)	0.083
Tumour size (mm)	≤30/>30	1.04 (0.71–1.51)	0.842
T status	T1–2/T3–4	1.27 (0.86–1.88)	0.233
Adjuvant therapy	Yes/No	1.81 (1.25–2.61)	**0.002**
Margin involvement	R0/R1	1.92 (1.24–2.97)	**0.003**
LNR (0)	N0	1	**< 0.001**
LNR	N1, ≤0.2	1.94 (1.30–2.88)	**0.001**
LNR	N1, >0.2	3.41 (1.96–5.95)	**< 0.001**
Albumin (g/L)	≥36/<36	1.56 (1.10–2.19)	**0.011**
CRP (mg/L)	≤5 />5	1.44 (1.03–2.02)	**0.036**
CEA (μg/L)	≤5/>5	1.60 (1.07–2.53)	**0.047**
CA19-9 (kU/L)	≤37	1	**0.015**
CA19-9 (kU/L)	38–554	1.09 (0.73–1.69)	0.633
CA19-9 (kU/L)	≥555	1.91 (1.18–3.08)	**0.008**

HR, hazard ratio; CI, confidence interval; LNR, lymph-node ratio

All these variables were included in one Cox proportional hazard model.

In multivariate analysis taking into account gender, age, T status, LNR, adjuvant therapy, tumour size, resection radicality, CA19-9, CEA, and GPS, a score of GPS 1 was not significantly associated with survival, whereas GPS 2 raised the HR significantly to 2.2 compared to HR for GPS 0 (*p* = 0.001) (Table 5). These results were similar to those of mGPS, with the same factors remaining statistically significant.

**Table 5:** Multivariate model considering GPS and tumour markers as well as clinically and statistically important patient characteristics (n = 189).

Factor			
	Description	HR (95% CI)	*p* value
Age (years)	<65/≥65	1.23 (0.80–1.60)	0.500
Sex	Male/female	1.36 (0.95–1.95)	0.096
Tumour size (mm)	≤30/>30	1.04 (0.72–1.50)	0.838
T status	T1-2/T3-4	1.32 (0.90–1.95)	0.159
Adjuvant therapy	Yes/No	1.84 (1.28–2.65)	**0.001**
Margin involvement	R0/R1	2.02 (1.32–3.10)	**0.001**
LNR (0)	N0	1	**< 0.001**
LNR	N1, ≤0.2	1.91 (1.29–2.85)	**0.001**
LNR	N1, >0.2	3.40 (1.97–5.87)	**< 0.001**
CEA (μg/L)	≤5/>5	1.46 (0.93–2.30)	0.100
CA19-9 (kU/L)	<555/≥555	1.82 (1.22–2.72)	**0.003**
GPS	0	1	**0.006**
GPS	1	1.21 (0.82–1.79)	0.333
GPS	2	2.18 (1.35–3.50)	**0.001**

HR, hazard ratio; CI, confidence interval; LNR, lymph-node ratio; GPS, Glasgow prognostic score

All these variables were included in one Cox proportional hazard model.

## Discussion

In this large retrospective study, we show that in surgically treated pancreatic ductal adenocarcinoma (PDAC), preoperatively increased levels of plasma CRP independently predict worse postoperative prognosis. Additionally, low serum albumin, elevated tumour markers CA19-9 and CEA, as well as a larger lymph-node ratio (LNR, N0 / ≤ 0.2 / > 0.2), no adjuvant chemotherapy, and R1 resection margin status were associated independently with a grim postoperative outcome.

The relationship of inflammation and cancer progression has intrigued scientists for the past two decades, yet the underlying reason for the unfavourable prognosis of cancer-related SIR remains partially unexplained [25,26]. Cancer-induced inflammation releases increased levels of circulating inflammatory cytokines such as interleukin 6 and TNFα, both of which initiate SIR. Cancer-related SIR correlates with symptoms associated with cancer such as fatigue, fever, weight loss, pain, and depression [27,28]. SIR may be related even to variability in the response to and the toxicity of cancer chemotherapy [25].

Our results support those of other studies concerning the assumption that SIR is detrimental to PDAC patients even if the tumour is resected radically [29–31]. Elevated CRP showed no significant association with those tumour-related factors typically associated with survival (tumour size, T status, LNR). This may suggest that SIR development is not directly connected to these tumour-related factors. Circulating acute-phase proteins can also increase due to other conditions more common than cancer, ones such as acute infections and auto-immune diseases. It is thus important to separate cancer-related SIR from these conditions, the ones which we took into account as our exclusion criteria. An advantage of our study is the detailed and reliable clinico-pathological information available from all patients, including medical history, tumour stage, and histological type.

One of the most validated prognostic scores based on laboratory variables is the Glasgow prognostic score (GPS) combining high CRP and low albumin [16]. In one large cohort study exploring several cancer types, hypoalbuminemia alone was associated with worse survival in some other cancer types but not with hepatopancreaticobiliary cancers, and their conclusion was that the modified GPS (mGPS), emphasizing elevated CRP, is a more universal prognostic score than is the GPS [17]. In our study, preoperative hypoalbuminemia was independently associated with worse survival, thus supporting the use of GPS instead of mGPS for resectable PDAC. It associated also with tumour size and T-status. Hypoalbuminemia as an independent prognostic factor in resectable PDAC has produced conflicting results, with some showing a similar association [29,32], but others showing contradictory results [33]. Cachexia and poor performance status are common in PDAC patients and they are associated with worse survival and hypoalbuminemia. However, all the patients in the present study were preoperatively evaluated to be fit for surgery (the Karnofsky Performance Status Scale of ≥ 60 [34]).

Besides CRP and hypoalbuminemia, elevation of the tumour markers CA19-9 and CEA correlated independently with worse prognosis [20,35]. Additionally, tumour-related factors are strong mediators in the prognostic evaluation of surgically treated PDAC [5,36]. Multivariate analysis showed that lymph-node ratio and resection margin status associated strongly with survival. T-status and tumour size were significant factors in univariate analysis but not in multivariate analysis. This might result from selection bias in our study, as patients with a resectable disease tend to have smaller tumours than do pancreatic cancer patients on average. Additionally, adjuvant therapy was associated with more favourable prognosis. The obvious prognostic disadvantage of the tumour-related factors is that they are available only during or after surgery. Increasing evidence of the prognostic value of preoperative CRP, albumin, and GPS support their utility for clinical decision-making in evaluation of postoperative survival, possibly combined with tumour markers. In fact, recent international guidelines have acknowledged the association between cancer-related SIR and PDAC survival, by recommending measurement of either mGPS or the neutrophil-to-lymphocyte ratio (NLR) in all patients considered for PDAC resection [37]. Our study supports the routine analysis of CRP and albumin preoperatively of all patients with PDAC considered for surgical treatment as a part of the general evaluation of prognosis. Further validation is necessary to make actual guidelines concerning their use in clinical practice. In light of our study elevated CRP combined with hypoalbuminemia and tumour markers might be helpful for selecting patients for surgery, especially in borderline cases. These results also raise the question whether patients with preoperative SIR would benefit from treatment with anti-inflammatory drugs or neoadjuvant therapy. This could provide a new and interesting research field.

A limitation of our retrospective study was some missing laboratory results and incomplete information in some pathology reports. Tumour grade in particular was seldom reported for earlier tumour samples and therefore underwent no analysis. Furthermore, because neutrophil and lymphocyte counts had no routine analysis, we were unable to determine NLR, recently proposed as a potential prognostic factor [14,33]. The present study is nonetheless one of the largest on the association of preoperative biomarkers with the prognosis of surgically treated PDAC.

In conclusion, this large retrospective study of patients with resectable PDAC shows that elevated CRP and hypoalbuminemia, as well as the GPS based on these parameters, were independent prognostic markers, indicating that preoperative cancer-related SIR is predictive of worse survival.
